# Dynamic 18F-FDG PET characterizes age-related changes in fitted ventricular FDG kinetics in humans

**DOI:** 10.1162/IMAG.a.1395

**Published:** 2026-10-13

**Authors:** Zeyu Zhou, Tianyun Zhao, He Wang, Aaron Carretero Benites, John Gardus, Qiuting Wen, Yang Feng, Christine DeLorenzo, Ramin Parsey, Chuan Huang

**Affiliations:** Radiology and Imaging Sciences, Emory University, Atlanta, GA, United States; Biomedical Engineering, Stony Brook University, Stony Brook, NY, United States; Biomedical Engineering, Georgia Institute of Technology and Emory University, Atlanta, GA, United States; Psychiatry and Behavioral Sciences, Stony Brook University, Stony Brook, NY, United States; Radiology and Imaging Sciences, Indiana University, Bloomington, IN, United States; Biostatistics, New York University, New York, NY, United States

**Keywords:** dynamic 18F-FDG PET, ventricular tracer kinetics, glymphatic-associated transport, aging, major depressive disorder, kinetic modeling

## Abstract

Ventricular cerebrospinal fluid (CSF) transport is an important component of intracranial solute movement and may contribute to glymphatic-associated transport pathways. However, clinically accessible methods for quantifying ventricular CSF tracer dynamics in humans remain limited. This study examined the potential of dynamic 18F-fluorodeoxyglucose (18F-FDG) positron emission tomography (PET) for quantifying ventricular CSF tracer kinetics as an indirect measure of CSF transport by evaluating its association with age and clinical factors. We analyzed baseline dynamic 18F-FDG PET data from participants with major depressive disorder enrolled in a prior clinical trial. Time–activity curves extracted from the lateral ventricles were modeled using a variant of the single-tissue compartment kinetic model to estimate kinetic parameters describing effective tracer entry into (*K_1_*) and net removal (*k_2_*) from the ventricular compartment. Associations with age and clinical factors were assessed using correlation and multiple linear regression analyses. Both *K_1_* (r = -0.700, p < 0.001) and *k_2_* (r = -0.712, p < 0.001) demonstrated strong negative correlations with age. In multivariable models adjusting for sex, ventricular volume, and depression severity, age remained a significant predictor of both parameters. Ventricular volume was also a significant predictor of both *K_1_* and *k_2_*, while *k_2_* additionally showed a significant association with depression severity. These findings suggest that dynamic 18F-FDG PET can characterize ventricular tracer kinetics without intrathecal contrast administration, revealing a robust age-dependent decline in both tracer entry and removal processes and supporting its potential utility for studying ventricular CSF transport dynamics in aging and disease.

## 1 Introduction

Interest in cerebrospinal fluid (CSF) dynamics has grown substantially following the discovery of the glymphatic system. The glymphatic pathway has been proposed to support intracranial solute movement and waste removal, with potential relevance to neural homeostasis. Alterations in glymphatic-associated transport have been implicated in a range of neurodegenerative disorders, including Alzheimer’s disease (AD), Parkinson’s disease (PD), and vascular dementia, where impaired clearance of metabolic waste may contribute to disease progression (Benveniste et al., 2021; Jessen et al., 2015). In addition, glymphatic-associated transport abnormalities have been linked to small vessel disease (Mestre et al., 2017), traumatic brain injury (Venkat et al., 2017), stroke-related impairment (Eide & Ringstad, 2015), and idiopathic normal pressure hydrocephalus (Ringstad et al., 2017). Although the precise relationship between ventricular CSF flow and glymphatic activity remains unclear, solute movement through the ventricular CSF space represents an important component of CSF circulation and contributes to the brain’s overall waste-handling processes (Ringstad et al., 2018; Taoka & Naganawa, 2020).

CSF is produced primarily by the choroid plexus located in the lateral, third, and fourth ventricles, with the lateral ventricles contributing the largest share of CSF production (Margetis et al., 2025). From the lateral ventricles, CSF flows through the ventricular system into the subarachnoid space, establishing the initial segment for intracranial circulation (Margetis & Baker, 2025). Because the lateral ventricles serve as both the primary site of CSF production and the starting point of its downstream distribution, characterizing solute transport within and from the lateral ventricles provides valuable insight into early stage CSF dynamics. Recent interventional studies targeting extracranial lymphatic drainage further support the clinical relevance of brain fluid clearance pathways, but direct imaging markers of ventricular CSF transport remain limited (X. Li et al., 2024).

Despite this growing interest, clinically practical techniques for characterizing ventricular CSF transport in humans remain limited. Current human imaging approaches primarily rely on magnetic resonance imaging (MRI) techniques, including dynamic contrast-enhanced MRI (DCE-MRI) with intrathecal tracers, phase-contrast MRI (PC-MRI), and diffusion-weighted imaging (DWI), each providing valuable insights into ventricular CSF dynamics (Taoka & Naganawa, 2020). DCE-MRI, while valuable for visualizing solute transport, often requires invasive intrathecal administration of gadolinium-based agents, posing practical and safety considerations (Eide & Ringstad, 2015). PC-MRI provides important velocity-based measures of pulsatile CSF flow but is less suited for slow, spatially distributed solute transport (Ringstad et al., 2017). Diffusion-based methods can provide indirect information about water diffusion and perivascular microstructure (Wright et al., 2024), but their relationship to bulk CSF solute transport remains indirect and sensitive to acquisition and modeling choices (Prasuhn et al., 2024). Emerging approaches such as relaxation-exchange MRI can quantify water exchange across the blood–CSF barrier, but do not directly capture solute transport dynamics within the ventricular compartment (Wu et al., 2025). These technical and practical challenges underscore the need for alternative imaging biomarkers that can quantify ventricular tracer dynamics without intrathecal contrast administration.

Positron emission tomography (PET) offers a fundamentally different approach by measuring tracer concentration over time. This allows quantification of net mass transport rather than instantaneous fluid velocity (de Leon et al., 2017; Y. Li et al., 2022; Schubert et al., 2019), making PET particularly well suited to study slow and spatially distributed tracer processes within the ventricular system. PET-derived kinetic measures can capture both tracer exchange into and removal from the ventricular compartment, integrating across multiple transport pathways and capturing physiologically meaningful changes that may not be apparent in velocity-based MRI modalities. Among PET tracers, 18F-fluorodeoxyglucose (18F-FDG), a glucose analog, presents a unique opportunity for ventricular CSF imaging, as preclinical MRI studies using glucose-based tracers have demonstrated links between CSF transport dynamics and neurodegenerative pathology in conditions such as AD and Huntington’s disease (Huang et al., 2020; Liu et al., 2023). Unlike receptor-binding PET tracers, 18F-FDG does not rely on ligand–receptor interactions, supporting assessment of tracer kinetics within the CSF space. Its wide clinical availability and established use further support its potential for translational applications.

In this study, we propose the use of dynamic 18F-FDG PET to quantify ventricular tracer kinetics as an indirect measure of solute transport within the ventricular CSF space. Our objective is to characterize how these ventricular tracer dynamics, including both effective tracer entry into and net removal from the ventricular compartment, vary with aging in a secondary analysis of a major depressive disorder (MDD) cohort. This work aims to evaluate the feasibility of 18F-FDG PET as a potentially clinically useful tool for characterizing ventricular CSF transport dynamics and glymphatic-associated transport, providing a foundation for future studies on neurodegenerative disease biomarkers and therapeutic interventions.

To our knowledge, this is the first human study to apply dynamic 18F-FDG PET with compartmental modeling to quantify age-related differences in ventricular tracer kinetics. Given that impaired CSF transport and bulk motion have been implicated in aging, AD, PD, traumatic brain injury, and normal-pressure hydrocephalus, a reproducible method to characterize ventricular CSF dynamics without intrathecal contrast administration is of broad translational relevance. This study lays the foundation for a scalable PET-based approach to studying ventricular CSF transport and glymphatic-associated transport that can be used across multiple neurological disorders.

## 2 Methods

### 2.1 Participants

This study is a secondary analysis of data originally acquired in a randomized clinical trial investigating cerebral glucose metabolism in major depressive disorder (Hill et al., 2021). A total of 85 participants (age range 18.1–64.5 years, mean ± standard deviation [SD]: 30.3 ± 13.9; 56 females) underwent baseline 18F-FDG PET imaging. The original study was approved by institutional review board, and all participants provided written informed consent in accordance with its guidelines.

Of the 85 baseline scans, 5 were excluded due to irrecoverable reconstruction issues, and additional 5 were removed due to severe image corruption from excessive motion. Three scans were also excluded due to abnormal blood glucose levels (outside 70–150 mg/dL). Two further scans were excluded due to the insufficient blood samples. This resulted in 70 participants being included in the baseline analysis cohort (Fig. 1).

**Fig. 1.**
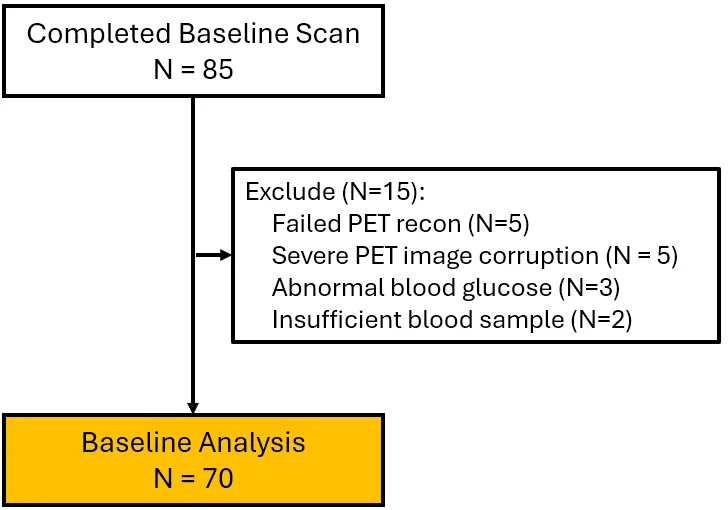
Participant flow diagram showing inclusion, exclusions.

### 2.2 PET/MR data acquisition

Each participant received an intravenous injection of 148–185 MBq (4–5 mCi) of 18F-FDG and was placed in a Siemens Biograph mMR PET/MR scanner for a 60-minute scan. PET data were acquired in list-mode format, then binned into 12 ten-second frames followed by 29 two-minute frames. Reconstructions were performed using Siemens E7 Tools with 75 iterations of the maximum-likelihood expectation maximization (MLEM) algorithm, resulting in a voxel size of 1.04 × 1.04 × 2.03 mm. This iteration number was selected based on contrast-to-noise ratio analysis, which plateaued around 75. The effective spatial resolution of the Siemens Biograph mMR is 4.3 mm FWHM at the center of the field of view (Delso et al., 2011). No post-reconstruction smoothing was applied and point spread function (PSF) modeling was not used. Concurrently, anatomical MR images were acquired using T1-weighted Magnetization Prepared Rapid Gradient Echo (MPRAGE) sequence with typical parameters: repetition/echo/inversion time = 2300/2.98/900 ms, flip angle = 9°, 1 mm isotropic resolution. MR-based attenuation correction was applied using the “Boston method” (Poynton et al., 2014). Participants were instructed to remain still and awake; no sedation was used. Arterial and/or venous blood samples were collected at multiple time points during the PET acquisition and used to measure plasma radioactivity for input-function calibration. During PET acquisition, arterial blood sampling was performed in a subset of participants with frequent early sampling followed by additional samples at approximately 20, 40, and 60 minutes post-injection. Venous blood samples were also obtained during the scan in some participants, including certain individuals who underwent arterial sampling. One or two discrete venous samples were collected during late phase of the scan, typically around 40 minutes and, when available, near 60 minutes post-injection.

### 2.3 Image processing

The 29 two-minute PET frames were averaged to generate a composite mean image, which served as the reference for motion correction. Each frame was rigidly realigned to this mean, and the process was repeated once to further reduce residual motion. The motion-corrected frames were then co-registered to each subject’s T1-weighted MR image using FSL v6.0.5.5 (Jenkinson et al., 2002). The 12 ten-second frames were assumed to have negligible motion and were assigned the same transformation estimated for the first 2-minute frame.

Each subject’s T1-weighted MPRAGE was processed by FreeSurfer v7.4.1 (Fischl, 2012) to produce an initial lateral ventricle mask. The mask was eroded by 1 mm in MRI space to reduce partial-volume contamination from periventricular tissue, choroid plexus, and vascular signal. To assess sensitivity to erosion level, the full pipeline was additionally repeated at 2 and 3 mm erosion. A 4 mm erosion condition was also evaluated but resulted in insufficient ventricular ROI volume in the majority of subjects, particularly younger participants with smaller ventricles, and was, therefore, excluded (Supplementary Section S5). Time–activity curves (TACs) were extracted from the eroded region of interest (ROI) across all dynamic frames for subsequent kinetic modeling and analysis. Additionally, periventricular region was extracted by iteratively dilating the ventricle ROI by 3 mm and then excluding the ventricles. Mean SUVs of the periventricular region were then obtained for all time points.

To assess whether residual choroid plexus signal influenced the fitted kinetic parameters, a choroid plexus sensitivity analysis was performed using an AI-based segmentation method (J. Li et al., 2025); results are reported in Supplementary Section S6.

### 2.4 Population-based arterial input function

To enable consistent quantification among samples and to accommodate cases without arterial sampling, we constructed a population-based arterial input function (pAIF). Arterial plasma input functions from participants with usable arterial sampling were first normalized by their area under the curve (AUC) from 0 to 60 minutes post-injection (Naganawa et al., 2020). Each normalized AIF was then fitted using the Feng plasma kinetic model (Feng et al., 1993):

$$C_{p}\left(t\right)=\{\begin{array}{ll} \;\;\;\;\;\;0 & if\;t<\tau \\ \left[A_{1}\left(t-\tau \right)-A_{2}-A_{3}\right]e^{-\lambda_{1}\left(t-\tau \right)}+A_{2}e^{-\lambda_{2}\left(t-\tau \right)}+A_{3}e^{-\lambda_{3}\left(t-\tau \right)} & if\;t\geq \tau \end{array}$$

where *A_i_* and λ*_i_* represent model coefficient and τ is a delay term. The fitted normalized curves were evaluated on a common grid and averaged across subjects to generate a mean normalized template curve. The averaged curve was subsequently refitted using the same Feng model to obtain the final pAIF (Naganawa et al., 2020).

### 2.5 Subject-specific scaling

For individual kinetic analysis, the template pAIF was scaled to each participant using late-phase plasma measurement. When arterial samples were available, arterial measures were preferentially used. In the absence of arterial sampling, venous plasma samples were used for scaling.

A single multiplicative scale factor was estimated by fitting the template pAIF to measured plasma concentrations at late time point (approximately 30–60 minutes post-injections).

To account for systematic differences between venous and arterial plasma concentrations in the late phase, subjects with paired arterial and venous samples were used to derive a venous-to-arterial correction factor. For participants with venous-only sampling, the scale factor was adjusted using the median arterial-to-venous ratio to approximate arterial-equivalent input functions.

### 2.6 Kinetic model

To model the dynamics of 18F-FDG in the lateral ventricles, we adapted a single-tissue compartment framework commonly used in dynamic FDG PET kinetic modeling to characterize ventricular tracer kinetics (Chung et al., 2025). A schematic representation of the model is shown in Figure 2. Because morphological erosion could not completely remove the choroid plexus and small intraventricular vessels, we assumed that a certain fraction of blood volume (*v_b_*) is present within the ventricles, and that this vascular component contributes directly to the measured standardized uptake value (SUV). In this model, *K_1_* represents the effective transfer of tracer from the plasma input into the measured ventricular compartment, while *k_2_* characterizes net removal of tracer from that compartment. *k_2_* should be considered an aggregate, model-derived tracer washout parameter that may be influenced by CSF transport but also by other physiological and technical factors, rather than a direct measure of CSF clearance. Although the choroid plexus likely contributes to ventricular tracer entry, *K_1_* should be interpreted as an effective input parameter rather than a direct measure of CSF production.

**Fig. 2.**
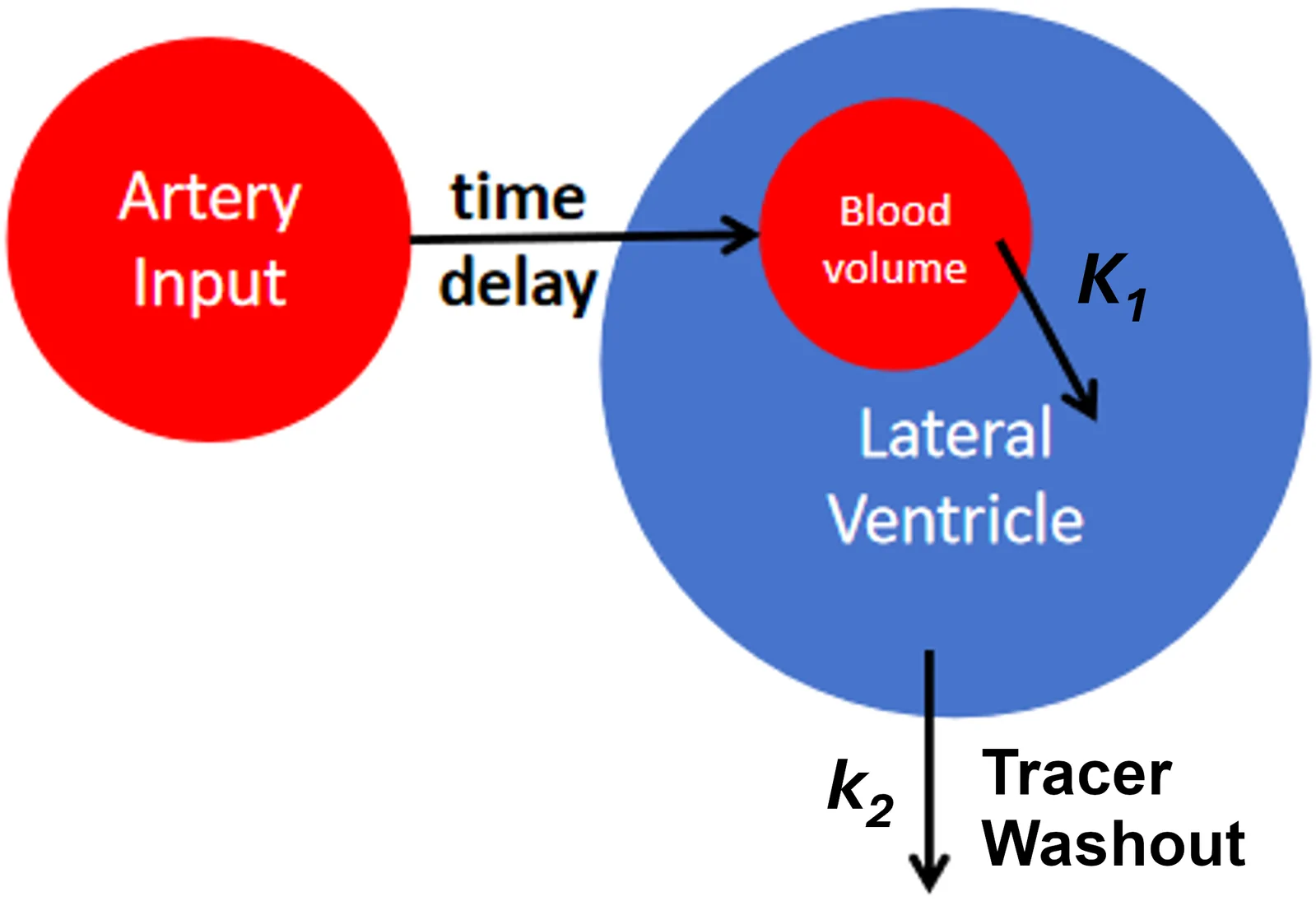
Schematic illustration of the ventricular compartment model. Ventricular PET activity is modeled as blood-volume spill-in and a single CSF compartment with effective input *K_1_* and effective removal *k_2_* driven by the delayed arterial input.

The impulse response function of the modified single-tissue compartment (S1TC) model is expressed as

$$R^{S1TC}\left(t\right)=v_{b}\delta \left(t\right)+K_{1}e^{-k_{2}t},$$

where δ*(t)* is the Dirac delta function. Predicted ventricular SUV time course is

$$Q^{S1TC}\left(t\right)=v_{b}C_{wb}\left(t-t_{d}\right)+K_{1}\int_{t_{d}}^{t}C_{p}\left(\tau -t_{d}\right)e^{-k_{2}\left(t-\tau \right)}d\tau ,$$

where *C_wb_(t)* and *C_p_(t)* are the whole-blood and plasma AIF, respectively. *t_d_* is the time delay between radiotracer arrival at the measured arterial input location and local tissue. For model simplicity, the same scaled population-based input function was used for both plasma input and vascular blood terms. This approximation is expected to have the greatest impact on the vascular spill-in component and should be considered when interpreting *v_b_*, *K_1_*, and *k_2_*. Under this formulation, *v_b_* remains part of the model mathematically but is partially confounded with AIF amplitude and, therefore, should be interpreted with physiological caution.

To illustrate the distinct contributions of *K_1_* and *k_2_* to the observed ventricular TAC shape, simulated time–activity curves were generated using the S1TC model with a representative population AIF, varying *K_1_* at fixed *k_2_* and *k_2_* at fixed *K_1_* across the cohort’s parameter range and are included in Supplement Section S4.

### 2.7 Statistical analysis

The primary outcome measures, *K_1_* and *k_2_*, were examined for normality using Shapiro–Wilk test and Q–Q plots. After outlier handling based on the interquartile range (IQR) method, both parameters were normally distributed and were, therefore, evaluated using Pearson correlation to assess associations with age.

To account for potential confounders, multiple linear regression models were constructed separately for *K_1_* and *k_2_*, incorporating age, lateral ventricle volume, baseline HDRS-17 score, and sex as predictors.

All statistical tests were two-tailed, with significance defined as p < 0.05. Analyses were performed using SPSS 31.0.0 and Python 3.10.13 with SciPy (version 1.11.3)

## 3 Results

### 3.1 Cohort characteristics

To investigate age-related differences in ventricular tracer kinetics in this MDD cohort, we analyzed 70 baseline FDG-PET scans. Demographic characteristics are summarized in Table 1. No significant sex differences were observed in baseline age, depression severity (HDRS-17), or lateral ventricle size.

**Table 1.** Baseline participant demographics.

Sex	N	Age	HDRS-17	Lateral Ventricle Size (cm^3^)
M	23	28.8 (11.8)	18.1 (5.0)	15.67 (6.64)
F	47	30.9 (15.3)	17.3 (4.4)	13.25 (5.84)
		p = 0.569	p = 0.492	p = 0.110

Values are expressed as mean (SD). Males and females were compared using independent two-sample t-test. HDRS-17: Hamilton Depression Rating Scale-17.

Kinetic parameters were estimated for all participants. Outliers were identified using the IQR method. No outliers were detected for *K_1_* (final N = 70), whereas three outliers were identified and excluded for *k_2_*, resulting in a final sample size of N = 67 for all *k_2_*-related analyses.

### 3.2 Age-related differences in ventricular FDG dynamics

Representative dynamic PET images and corresponding lateral ventricle TACs with S1TC model fits are shown in Figure 3. In both participants, tracer activity within the ventricles increased over time; however, clear age-related differences in temporal dynamics were observed. The younger participant demonstrated faster early accumulation and earlier plateauing, whereas the older participant showed slower accumulation with continued signal increase throughout the scan. These patterns were consistent with reduced *K_1_* and *k_2_* values in the older participant.

**Fig. 3.**
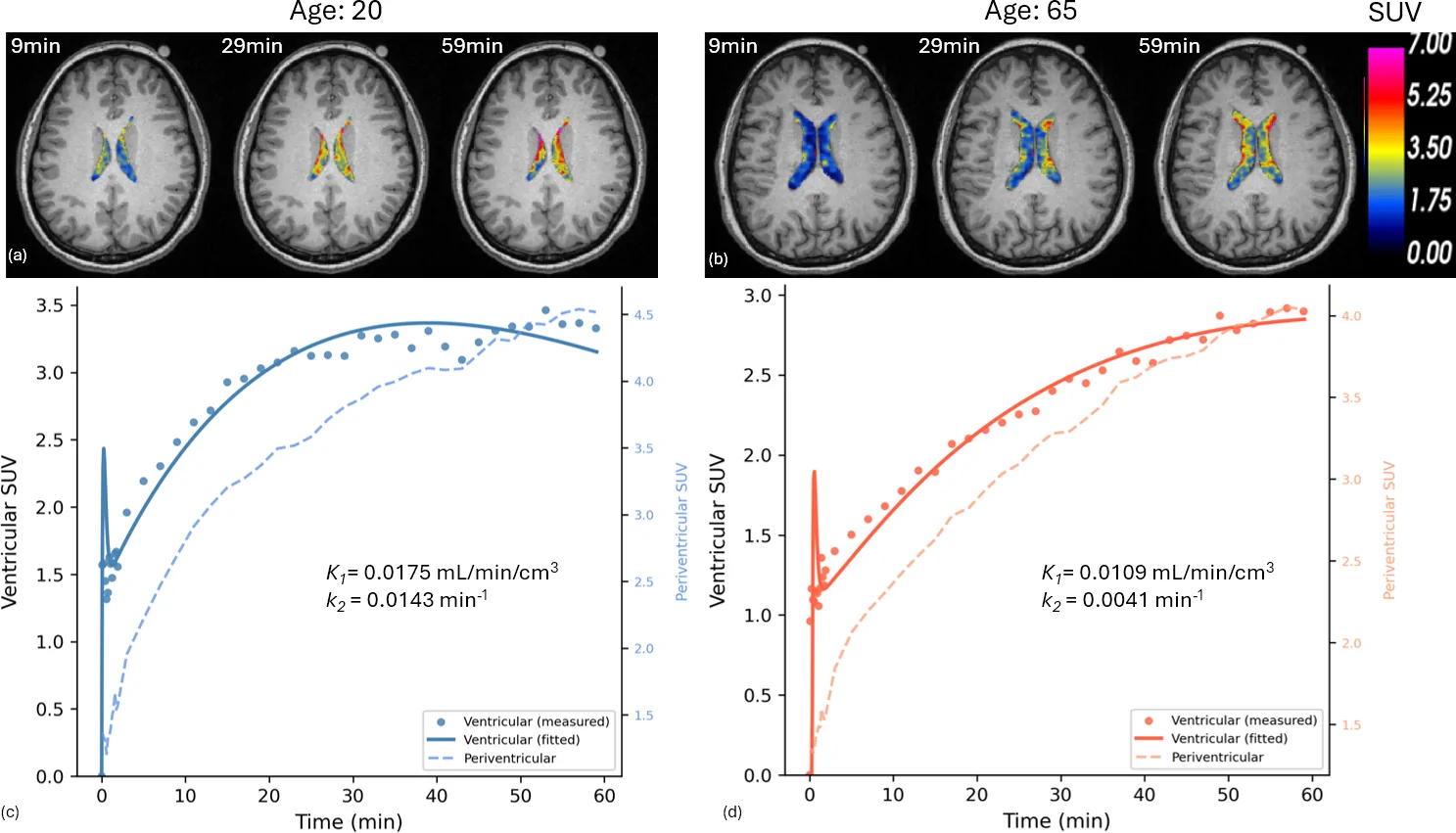
Representative dynamic PET/MR images (a, b) and corresponding lateral ventricle TACs (c, d) with S1TC model fits for a younger (age 20 years, ventricular volume 11.1 cm³) and older (age 65 years, ventricular volume 19.5 cm³) participant. The younger participant shows faster early accumulation and earlier plateauing, whereas the older participant shows slower accumulation with continued increase over time, lower *K_1_* and *k_2_* values. Dashed lines show the periventricular TAC (right y-axis).

To visualize these patterns across the cohort, lateral ventricle TACs were normalized by their 2-norm (Fig. 4a) and fitted using the kinetic model (Fig. 4b). A clear age-related gradient was observed: older participants (depicted in red) exhibited flatter, continuously rising curves, whereas younger individuals (blue) showed earlier plateaus, consistent with age-related differences in tracer exchange dynamics.

**Fig. 4.**
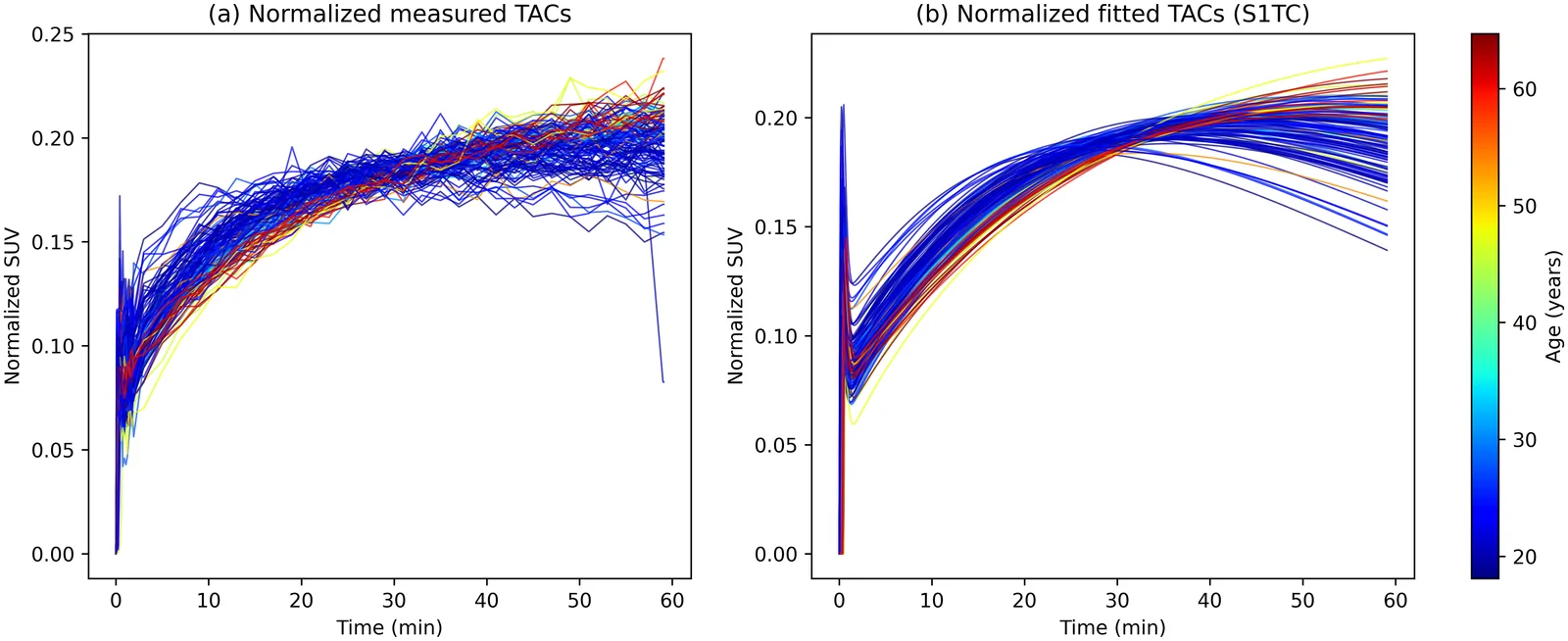
(a) Normalized lateral ventricle TACs and (b) corresponding S1TC model fits for all participants (N = 70), color-coded by age.

Building on the observed cohort-level patterns, we next quantified the relationship between kinetic parameters and age. *K_1_* and *k_2_* were both normally distributed (Shapiro–Wilk test, p > 0.05) after outlier handling and were, therefore, evaluated using Pearson correlation. *K_1_* showed a strong negative correlation with age (r = -0.700, p < 0.001), and *k_2_* similarly demonstrated a strong negative correlation (r = -0.712, p < 0.001) (Fig. 5). Both parameters showed significant age-related associations at the primary erosion level, and these associations remained significant across erosion depths, as described below.

**Fig. 5.**
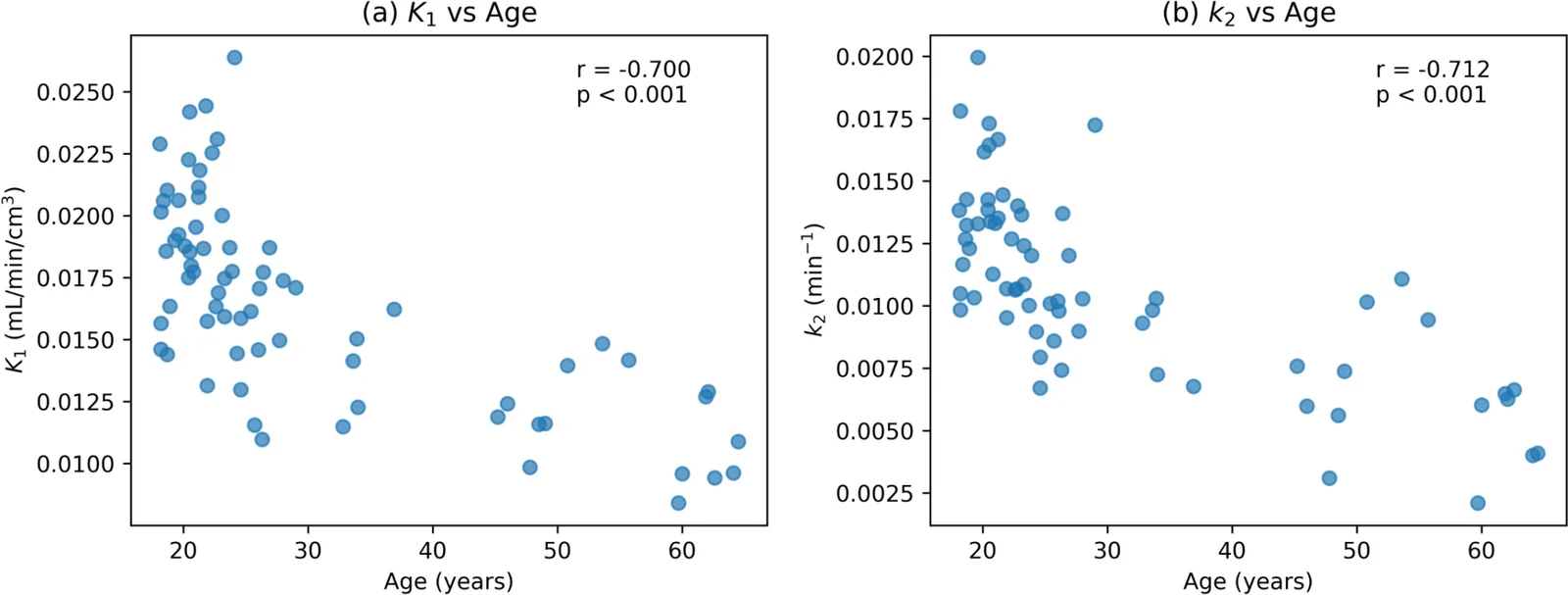
Scatter plot of fitted parameters (a) *K_1_* and (b) *k_2_* against age. Both parameters show strong negative correlation with age (Pearson correlation). *k_2_* analysis excludes IQR-defined outliers (N = 67).

To further account for potential confounding effects, multivariable linear models were constructed including age, ventricular volume, sex, and depression severity (HDRS-17) (Table 2). For *K_1_*, age (β = -0.519, p < 0.001) and ventricular volume (β = -0.429, p < 0.001) were significant predictors, whereas HDRS-17 (β = 0.045, p = 0.538) and sex were not significant predictors. In contrast, *k_2_* was significantly associated with age (β = -0.511, p < 0.001), ventricular volume (β = -0.422, p < 0.001), and HDRS-17 (β = 0.208, p = 0.004), but not with sex (β = 0.012, p = 0.865). These findings suggest that both parameters are strongly influenced by age and ventricular volume, while *k_2_* shows an additional cross-sectional association with depression severity. Additional derived metrics, including the ratio *K_1_*/*k_2_* and *k_2_* multiplied by ventricular volume, were explored and are presented in the Supplementary Materials.

To assess robustness to partial-volume control, analyses were repeated at 2 and 3 mm erosion levels. The Pearson correlation between *K_1_* and age was r = −0.700, −0.715, and −0.707 at 1, 2, and 3 mm erosion, respectively, remaining stable across all three levels. The Pearson correlation between *k_2_* and age was r = −0.712, −0.678, and −0.630 (all p < 0.001), remaining significant but showing progressive attenuation with increasing erosion level, consistent with increasing *K_1_–k_2_* coupling at smaller ROI sizes. Pre- and post-erosion ventricular volumes are summarized in Table 3.

**Table 2.** Multiple linear regression results for *K_1_* and *k_2_* across erosion level.

Erosion		1 mm		2 mm		3 mm	
*K_1_* Model Fit		${\text{R}}^{2}$ = 0.663	p < 0.001	${\text{R}}^{2}$ = 0.691	p < 0.001	${\text{R}}^{2}$ = 0.684	p < 0.001
		β	p-Value	β	p-Value	β	p-Value
*K_1_*	Age (year)	-0.519	<0.001	-0.527	<0.001	-0.514	<0.001
	Lateral Ventricle Size (cm^3^)	-0.429	<0.001	-0.443	<0.001	-0.453	<0.001
	HDRS-17	0.045	0.538	0.053	0.447	0.051	0.470
	Sex	-0.070	0.355	-0.056	0.441	-0.050	0.493
*k_2_* Model Fit		${\text{R}}^{2}$ = 0.705	p < 0.001	${\text{R}}^{2}$ = 0.635	p < 0.001	${\text{R}}^{2}$ = 0.603	p < 0.001
*k_2_*	Age (year)	-0.511	<0.001	-0.480	<0.001	-0.433	<0.001
	Lateral Ventricle Size (cm^3^)	-0.422	<0.001	-0.426	<0.001	-0.443	<0.001
	HDRS-17	0.208	0.004	0.155	0.046	0.160	0.049
	Sex	0.012	0.865	0.081	0.303	-0.045	0.584

Covariates included age, lateral ventricle size, HDRS-17 score, and sex.

β: standardized beta coefficient. HDRS-17: Hamilton Depression Rating Scale-17.

**Table 3.** Pre- and post-erosion lateral ventricular volumes at 1, 2, and 3 mm erosion levels.

	Pre-erosion	1 mm	2 mm	3 mm
Mean ± SD (cm^3^)	13.98 ± 6.18	8.25 ± 4.92	4.28 ± 3.59	1.93 ± 2.28
Median (cm^3^)	11.76	6.16	2.67	0.83
Range (cm^3^)	5.62–33.08	2.16–24.48	0.46–17.35	0.03–11.40

A choroid plexus sensitivity analysis confirmed that removal of choroid plexus voxels from the ventricular ROI produced negligible changes in both *K_1_* and *k_2_* across all erosion levels (Supplementary Section S6), supporting the adequacy of the blood volume term in accounting for residual vascular signal.

### 3.3 Association and independence of *K_1_* and *k_2_*

We next examined the relationship between *K_1_* and *k_2_*. A strong positive correlation was observed between the two parameters (r = 0.805, p < 0.001) (Fig. 6), indicating that individuals with higher *K_1_* also tended to exhibit higher *k_2_* values. This strong coupling is expected because both parameters are estimated from the same ventricular TAC, but it does not by itself indicate that the age association of *k_2_* is secondary to *K_1_*.

**Fig. 6.**
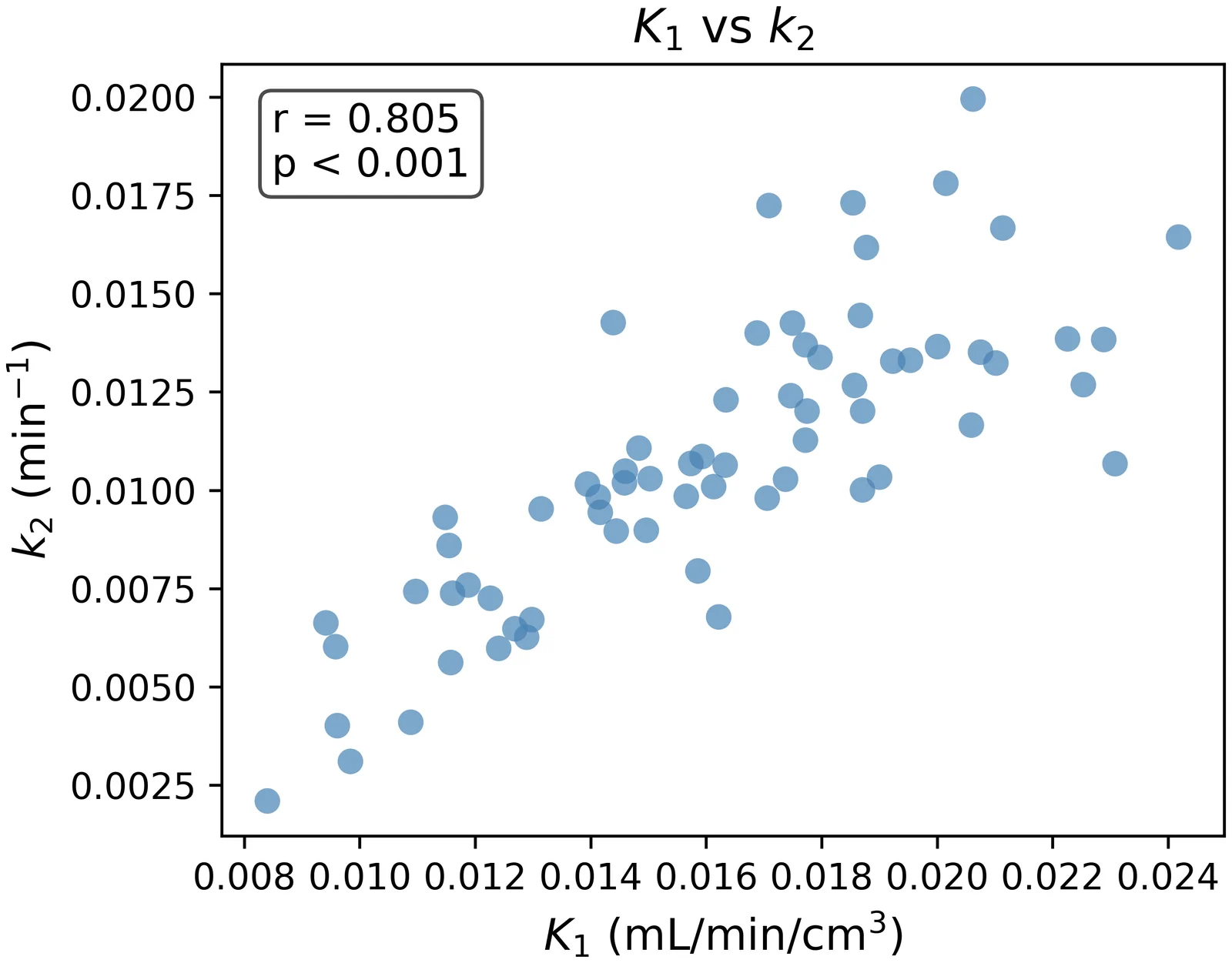
Scatter plot of fitted parameters *K_1_* versus *k_2_*. A strong positive correlation was observed between the two parameters (Pearson correlation, r = 0.805, p < 0.001). Analysis excludes IQR-defined *k_2_* outliers (N = 67).

To determine whether the associations with age for *K_1_* and *k_2_* were independent of each other, we constructed two multivariable regression models. In the first model, *k_2_* was treated as the dependent variable, and age, *K_1_*, ventricular volume, sex, and HDRS-17 were included as predictors. The overall model was significant (R^2^ = 0.748, p < 0.001), and age remained a significant predictor of *k_2_* (β = -0.296, p < 0.01) after accounting for *K_1_*. In the second model, *K_1_* was treated as the dependent variable, and age, *k_2_*, ventricular volume, sex, and HDRS-17 were included as predictors. Similarly, the overall model was significant (R^2^ = 0.769, p < 0.001), and age remained a significant predictor of *K_1_* (β = -0.346, p < 0.001) after accounting for *k_2_*. These results indicate that, at the primary erosion level, *K_1_* and *k_2_* are related but not redundant, and both contribute information about age-related differences in ventricular FDG kinetics.

## 4 Discussion

### 4.1 Age-related alterations in ventricular CSF tracer kinetics

These findings provide in vivo evidence that ventricular FDG-derived tracer dynamics are strongly influenced by aging. In our cohort, both *K_1_* and *k_2_* demonstrated robust negative associations with age (*K_1_*: r = −0.700, p < 0.001; *k_2_*: r = −0.712, p < 0.001), indicating that older individuals exhibit reduced effective tracer entry into the ventricular compartment and slower subsequent tracer removal. The comparable strength of these correlations suggests that aging affects multiple components of ventricular tracer kinetics rather than a single isolated process.

### 4.2 Relationship and interpretation of *K_1_* and *k_2_*

Given that *K_1_* and *k_2_* both demonstrated similar age-related declines, an important question is whether these parameters reflect distinct aspects of ventricular tracer kinetics or represent a shared underlying process. In this cohort, *K_1_* and *k_2_* were strongly positively correlated (r = 0.805, p < 0.001) across individuals, indicating that they capture related aspects of ventricular tracer kinetics. This coupling strengthened with increasing erosion level, consistent with greater signal homogeneity at smaller ROI sizes.

However, this relationship did not reflect simple redundancy between parameters. At the 1 mm level, both parameters retained independent age associations after mutual adjustment, suggesting they capture partially distinct aspects of ventricular tracer kinetics. Importantly, erosion sensitivity analyses revealed that *k_2_* remained strongly associated with age across erosion depths in both correlation and covariate-adjusted regression analyses, but its independent association after mutual adjustment with *K_1_* was attenuated at deeper erosion levels. This pattern likely reflects increasing coupling between *K_1_* and *k_2_* as the ROI becomes smaller and more spatially restricted, reducing the ability of the model to separate tracer entry and washout components. Thus, the attenuation of *k_2_* in mutual-adjustment models should be interpreted as reduced parameter separability rather than absence of an age-related *k_2_* effect. Taken together, these findings indicate that both *K_1_* and *k_2_* are strongly age-associated parameters of ventricular FDG kinetics, with *k_2_* remaining particularly relevant as a tracer washout parameter.

An additional consideration in interpreting the erosion analyses is partial-volume contamination from surrounding brain tissue. The PET data were acquired on a Siemens Biograph mMR scanner with an effective spatial resolution of approximately 4.3 mm FWHM; therefore, morphological erosion cannot fully eliminate spill-in from adjacent periventricular tissue. This is particularly relevant for dynamic FDG PET because activity in the surrounding brain parenchyma is substantially higher than that in ventricular CSF and remains elevated during the late dynamic frames. Consequently, the measured ventricular TAC may reflect both tracer kinetics within the ventricular compartment and residual contributions from adjacent tissue.

Importantly, the associations of both *K_1_* and *k_2_* with age remained significant across progressively eroded ventricular ROIs, although the *k_2_* association showed greater attenuation with increasing erosion depth. Because erosion reduces the contribution of boundary voxels, the persistence of these associations supports their robustness to ROI definition and to partial reduction of boundary-related contamination. However, progressively deeper erosion also reduces the number of ventricular voxels and measurement precision; therefore, the attenuation of the *k_2_* association cannot be attributed specifically to removal of partial-volume effects. Overall, these findings support *k_2_* as a useful model-derived descriptor of effective ventricular tracer removal, while its absolute value and physiological specificity remain potentially influenced by residual partial-volume effects and periventricular tracer kinetics.

### 4.3 Structural and clinical correlates of ventricular kinetic parameters

To further assess whether the observed age-related changes in *K_1_* and *k_2_* were influenced by structural or clinical factors, multivariable models (Table 2) were constructed including age, ventricular volume, sex, and depression severity (HDRS-17). Within this framework, both *K_1_* and *k_2_* remained significantly associated with age (*K_1_*: β = -0.519, p < 0.001; *k_2_*: β = -0.511, p < 0.001), indicating that the observed age-related alterations in ventricular tracer kinetics are not fully explained by these covariates.

Ventricular volume showed a significant association with both *K_1_* and *k_2_* (*K_1_*: β = -0.429, p < 0.001; *k_2_*: β = -0.422, p < 0.001), suggesting that structural factors contribute to variability in ventricular tracer kinetics. Because *k_2_* is a rate constant, its association with ventricular volume may partly reflect the dependence of concentration washout on compartment size rather than a purely physiological reduction in absolute tracer removal. In this context, the product of *k_2_* and ventricular volume may provide a complementary estimate of absolute ventricular tracer removal, helping distinguish compartment-size effects from changes in tracer removal capacity. However, although ventricular volume increases with age (Carmichael et al., 2007), the effect of age on both *K_1_* and *k_2_* remained significant after accounting for ventricular size, indicating that the observed changes in ventricular tracer kinetics cannot be explained solely by structural enlargement.

In contrast, only *k_2_* demonstrated an additional association with HDRS-17 (β = 0.208, p = 0.004), with higher symptom burden associated with higher *k_2_* values, which should not be interpreted as evidence of enhanced glymphatic function, whereas *K_1_* was not significantly related to depression severity. This association was consistent across erosion levels, suggesting it is not attributable to partial-volume contamination and may reflect a genuine relationship between depression severity and ventricular tracer removal dynamics. The positive association between HDRS-17 and *k_2_* may indicate that depression severity is associated with altered ventricular tracer exchange or removal dynamics. Because *k*_2_ reflects net tracer removal from the measured ventricular compartment, it may be influenced by multiple processes, including periventricular exchange, blood–CSF barrier transport, choroid plexus-related tracer handling, and downstream CSF washout. The lack of robust longitudinal prediction of symptom improvement suggests that the PET-HDRS relationship is more consistent with a cross-sectional state association than a treatment–response biomarker. Sex was not significantly associated with either parameter.

Together, these findings support the interpretation that age-related alterations in ventricular tracer kinetics reflect processes beyond gross anatomical variation, with additional, modest modulation of tracer removal dynamics by clinical state.

### 4.4 Mechanistic implications of altered ventricular tracer dynamics

The observed age-related reductions in both *K_1_* and *k_2_* indicate that aging affects multiple components of ventricular tracer kinetics, encompassing both effective tracer entry into and net removal from the ventricular compartment. Within the model framework, *K_1_* reflects effective tracer transfer from plasma into the measured ventricular compartment, whereas *k_2_* should be interpreted as an effective rate constant describing net tracer removal from the measured ventricular compartment. This parameter may reflect multiple processes, including ventricular mixing, periventricular exchange, blood–CSF barrier transport, and downstream CSF washout.

The age-related decline in *k_2_* suggests slower ventricular tracer removal with aging, consistent with altered ventricular CSF transport dynamics. However, *k_2_* is an effective kinetic parameter rather than a direct measurement of CSF flow velocity or brain-wide glymphatic transport. Accordingly, reductions in *k_2_* may arise from changes in ventricular mixing, periventricular exchange, blood–CSF barrier transport, downstream CSF washout, or combinations of these processes. The persistence of age-related associations after accounting for structural and clinical factors supports a physiological, rather than purely anatomical, basis for these alterations.

The reduction in *K_1_* with age suggests decreased efficiency of tracer entry into ventricular CSF. While the choroid plexus, a major site of CSF production and blood–CSF exchange, likely contributes, *K_1_* represents an effective, AIF-driven input term reflecting tracer transfer from plasma into the ventricular CSF compartment, and may incorporate multiple pathways beyond choroid plexus-mediated exchange. Thus, *K_1_* should be interpreted as a composite measure of tracer entry into the ventricular compartment rather than a direct index of a single physiological interface.

These observations are broadly consistent with prior studies demonstrating age-related alterations in CSF-related transport processes, including impaired CSF–interstitial fluid exchange in preclinical models (Kress et al., 2014), reduced water exchange across the blood–CSF barrier measured using MRI in humans (Wu et al., 2025), and declines in interstitial fluid transport with aging (Suzuki et al., 2024). While these measures reflect different aspects of fluid and solute transport than the ventricular FDG kinetics assessed here, they provide complementary evidence for broader age-related disruptions in brain transport dynamics.

Consistent with these interpretations, the temporal behavior of ventricular TACs provides qualitative support for altered late-phase tracer removal with aging. In particular, the continued increase in ventricular signals at later time points, especially in older individuals, suggests a slower approach to equilibrium and reduced effective washout, consistent with lower *k_2_* values. This delayed late-phase behavior also indicates that extended acquisition durations may improve the stability of *k_2_* estimation in aging populations.

The early phase ventricular signal may contain additional information related to blood–CSF barrier transport, similar to dynamic glucose-enhanced MRI studies. However, the brief 10-second early frames in this study yield low photon counts and high statistical noise, which combined with the small eroded ROI and rapidly evolving bolus dynamics during this window limits reliable extraction of early phase metrics. This remains a promising direction for future studies employing acquisition protocols optimized for early temporal resolution.

The S1TC model applied here does not separately account for tracer transport through brain parenchyma, which may contribute to the measured ventricular signal through periventricular exchange pathways. The periventricular TACs shown in Figure 3 illustrate that FDG activity in the periventricular parenchyma is substantially higher than ventricular signal and continues to rise throughout the acquisition window, providing qualitative evidence that both compartments undergo dynamic changes. Whether the observed age-related changes in *K_1_* and *k_2_* specifically reflect glymphatic or CSF transport processes, as opposed to changes in periventricular parenchymal exchange, cannot be resolved with the current model. Future studies employing higher spatial resolution, acquisition protocols optimized for ventricular CSF sampling, and multi-compartment models that explicitly account for parenchymal exchange pathways will be needed to disentangle these contributions.

### 4.5 Implications for CSF transport imaging with FDG PET

The present findings highlight the utility of dynamic 18F-FDG PET for characterizing ventricular tracer kinetics as an indirect biomarker of ventricular CSF transport and glymphatic-associated clearance. Unlike MRI-based approaches that primarily assess fluid motion or diffusion properties, PET captures the time-dependent distribution and retention of solutes, enabling assessment of both effective tracer entry into and net removal from the ventricular compartment through the parameters *K_1_* and *k_2_*.

From a physiological perspective, PET provides a mass-transport view of CSF-related dynamics rather than a direct measure of fluid velocity. As a result, PET-based measures may detect alterations in overall transport efficiency even in situations where flow velocities appear preserved. This complementary perspective is particularly relevant for studying age-related changes in brain transport processes, where multiple contributing mechanisms may be involved.

Compared with existing MRI-based techniques, PET offers a complementary methodological perspective. PC-MRI provides valuable velocity-based measures of pulsatile CSF flow but is less suited to slow, multidirectional solute transport within the ventricular system (Ringstad et al., 2017). DWI provides measures of molecular diffusion and indirect information about fluid motion and perivascular microstructure (Wright et al., 2024), but does not directly quantify net solute transport over time. Emerging approaches such as REXI can quantify water exchange across the blood–CSF barrier, but do not model the time-resolved distribution of solutes within the ventricular compartment (Wu et al., 2025). DCE-MRI approaches require intrathecal administration of contrast agents and are, therefore, more invasive and less practical for routine use (Eide & Ringstad, 2015).

In contrast, 18F-FDG PET is widely available and relies on standard intravenous administration, making it feasible for both research and clinical applications without intrathecal contrast administration. The relatively long half-life of 18F enables extended acquisition periods, allowing reliable estimation of slow kinetic processes such as ventricular tracer exchange and removal. The relatively stable late-phase ventricular signal provided by FDG further supports characterization of slow transport processes, which are critical for estimating parameters such as *k_2_*. In addition, the absence of specific receptor binding simplifies kinetic interpretation compared with other PET tracers that exhibit complex binding behavior.

The choice of tracer also influences the ability to characterize ventricular transport dynamics. PET tracers with specific binding, such as amyloid-β (Schubert et al., 2019) and tau radioligands (Y. Li et al., 2022), exhibit complex kinetics that can complicate the interpretation of ventricular signals, particularly at later time points. In contrast, 18F-FDG does not involve specific binding within the CSF, enabling more reliable estimation of both tracer exchange and removal processes. While freely diffusible tracers such as 15O-H_2_O (Suzuki et al., 2024) avoid binding effects, their short half-lives limit practical imaging windows and reduce sensitivity to slow ventricular dynamics. These considerations further support the use of FDG as a practical tracer for assessing ventricular transport processes.

Together, these features position dynamic FDG PET as a practical and complementary approach for investigating CSF-related transport dynamics, with potential applications in studying aging and disease-related alterations in brain transport processes.

### 4.6 Modeling considerations

The ventricular tracer kinetics in this study were characterized using a variant of the S1TC model, adapted from compartmental approaches commonly used in dynamic FDG PET kinetic modeling (Chung et al., 2025). Within this framework, the parameters *K_1_* and *k_2_* provide estimates of effective tracer exchange into and net removal from the ventricular compartment, respectively. This model enables a simplified and interpretable representation of ventricular tracer behavior over time.

As with any compartmental approach, the S1TC model involves several simplifying assumptions. In particular, the lateral ventricle is treated as a single, well-mixed compartment, and the estimated parameters reflect net transport processes rather than explicitly resolving spatial heterogeneity, mixing effects, or potential reflux pathways. These assumptions are reasonable for capturing late-phase tracer behavior but may not fully represent the underlying complexity of CSF dynamics.

Accordingly, the parameters *K_1_* and *k_2_* should be interpreted as aggregate descriptors of ventricular tracer kinetics rather than direct measurements of specific physiological mechanisms. While this modeling approach provides a useful framework for quantifying age-related differences in ventricular tracer dynamics, future studies incorporating more detailed spatial or multi-compartment models may further refine the characterization of CSF transport processes.

### 4.7 Limitations

Several limitations should be considered when interpreting these findings. First, this study is cross-sectional and, therefore, the observed associations with age do not directly reflect within-subject longitudinal changes. Longitudinal studies will be necessary to determine whether the observed alterations in *K_1_* and *k_2_* represent progressive changes over time.

Second, the *k_2_* parameter has not been independently validated against established direct measures of CSF dynamics. As an aggregate, model-derived tracer washout parameter, its physiological specificity remains to be established in future studies incorporating concurrent reference measurements of CSF flow or transport. Relatedly, test–retest reproducibility of *K_1_* and *k_2_* was not assessed, as the available data were derived from a retrospective clinical dataset with longitudinal changes in clinical status and ventricular structure, and, therefore, do not represent true repeat measurements. Assessment of reproducibility will require a dedicated dataset acquired under stable conditions.

Estimation of ventricular tracer kinetics relies on model-based fitting of dynamic PET data and is subject to assumptions inherent to the S1TC framework. In particular, the ventricular compartment is treated as a single, well-mixed space, and the estimated parameters reflect net transport processes rather than explicitly resolving spatial heterogeneity, mixing effects, or reflux pathways. The population-based AIF approach reduces the need for complete arterial sampling but may introduce uncertainty in individual kinetic estimates, particularly when arterial sampling is unavailable and late venous samples are used for subject-specific scaling. Dynamic PET kinetic modeling depends on the arterial input function, and population-based or estimated input functions can introduce parameter uncertainty despite careful validation and scaling.

While morphological erosion and choroid plexus sensitivity analyses support the robustness of the primary findings, partial-volume control remains incomplete given the 4.3 mm FWHM spatial resolution of the scanner. Residual spill-in from periventricular brain tissue, choroid plexus, and small intraventricular vessels cannot be fully excluded, and may influence the measured ventricular TAC and fitted kinetic parameters.

In addition, estimation of kinetic parameters, particularly *k_2_*, depends on accurate characterization of late-phase TAC behavior. In subjects exhibiting prolonged ventricular signal increase without a clear plateau within the 60-minute window, incomplete sampling of the terminal phase may affect parameter stability and introduce uncertainty in model fitting. This limitation is particularly relevant in older individuals, where delayed tracer washout may require longer acquisition durations for robust estimation.

Furthermore, while FDG PET provides a measure of tracer transport over time, it does not directly quantify CSF flow, CSF production, or brain-wide glymphatic transport, nor does it distinguish between specific contributing physiological mechanisms. Accordingly, PET-derived kinetic parameters should not be interpreted as direct measurements of individual transport pathways.

Finally, the study cohort consisted of individuals with MDD, which may limit the generalizability of these findings to healthy aging or other neurological populations. Although depression severity showed a cross-sectional association with *k*_2_, the extent to which clinical state influences ventricular tracer kinetics remains to be further investigated in broader populations.

## 5 Conclusion

This study supports the feasibility of dynamic 18F-FDG PET for quantifying ventricular tracer kinetics in humans without intrathecal contrast administration. In this adult MDD cohort, both effective tracer entry into the ventricular compartment (*K_1_*) and net tracer removal (*k_2_*) declined with age, consistent with age-related alterations in ventricular CSF transport dynamics. Within this kinetic framework, both *K_1_* and *k_2_* showed strong age-related associations. *K_1_* was more stable in mutual-adjustment analyses across erosion depths, whereas *k_2_* remained strongly age associated and may provide a more physiologically informative marker of ventricular tracer washout. Future studies with optimized spatial resolution, longer dynamic acquisition, and reference measures of CSF transport will be needed to further establish the specificity and reproducibility of *k_2_*. Unlike velocity- or diffusion-based MRI methods, 18F-FDG PET provides a mass-transport-based estimation of solute dynamics, offering a complementary perspective on ventricular transport processes. These findings position dynamic 18F-FDG PET-derived kinetic parameters as indirect biomarkers for investigating ventricular CSF transport and glymphatic-associated transport, with potential applications in aging research, early identification of altered transport dynamics, longitudinal monitoring, and evaluation of interventions that may influence CSF-related pathways.

## Supplementary Material

Supplementary Material

## Data Availability

The datasets used and/or analyzed during the current study are available from the corresponding author on reasonable request with formal data-sharing agreement and local ethics committee’s approval.
