# Supplementary material for: Dynamic 18F-FDG PET characterizes age-related changes in fitted ventricular FDG kinetics in humans
Source: Imaging Neurosci (Camb). 2026 Oct 13;4:IMAG.a.1395. doi: 10.1162/IMAG.a.1395 (PMC13662652; doi:10.1162/IMAG.a.1395)
Supplement: Supplementary file 1 [file IMAG.a.1395_supp.pdf]

Supplement

Sensitivity analyses were performed to evaluate the stability of the main findings under different assumptions. These included analyses with all subjects retained, including previously excluded outliers, as well as analyses using alternative derived metrics, including K_1/k_2 and k_2 × ventricular volume.

S1. Analysis with All Subjects Retained

To assess the impact of outlier exclusion, analyses were repeated with all subjects included, without removing IQR-defined outliers. Under these conditions, k_2 no longer satisfied normality assumptions (Shapiro-Wilk test, $p < 0.001$) and was therefore evaluated using Spearman correlation. The correlation between k_2 and age remained significant ($\rho = -0.717$, $p < 0.001$), consistent with the primary analysis.

In multivariable models adjusting for ventricular volume, sex, and HDRS-17, age remained a significant predictor of k_2 ($\beta = -0.465$, $p < 0.001$), while ventricular volume also showed a significant association ($\beta = -0.374$, $p < 0.001$). In contrast, HDRS-17 and sex were not significant predictors.

To further examine the relationship between K_1 and k_2, separate multivariable models were constructed. In the model with k_2 as the dependent variable and K_1 included as a predictor, the association between age and k_2 was attenuated and no longer statistically significant ($\beta = -0.077$, $p = 0.410$). In contrast, in the model with K_1 as the dependent variable and k_2 included as a predictor, the association between age and K_1 remained significant ($\beta = -0.262$, $p < 0.001$). These findings indicate that inclusion of the outlier subjects does not materially alter the overall association between k_2 and age, but it does affect the mutual-adjustment model, in which the age effect for k_2 no longer remains significant after accounting for K_1.

S2. Analysis Using K_1/k_2

To further evaluate the stability of the main findings under an alternative parameter representation, the ratio K_1/k_2 was examined as a derived metric. The distribution of K_1/k_2 did not satisfy normality assumptions and was therefore evaluated using Spearman correlation. K_1/k_2 showed a significant association with age ($\rho = 0.382$, $p < 0.001$).

In multivariable models adjusting for ventricular volume, sex, and HDRS-17, both age ($\beta = 0.394$, $p < 0.001$) and HDRS-17 ($\beta = -0.232$, $p < 0.5$) were significant predictors of K_1/k_2, whereas sex was not significant. Ventricular volume showed a trend-level association ($\beta = 0.220$, $p = 0.064$).

S3. Analysis Using k_2 × Ventricular Volume

To further evaluate the influence of ventricular volume on the observed associations, the product k_2 × ventricular volume was examined as an alternative representation. The distribution of k_2 × ventricular volume satisfied normality assumptions. The correlation between k_2 × ventricular volume and age was significant ($r = -0.353$, $p < 0.01$).

In multivariable models adjusting for ventricular volume, sex, and HDRS-17, age remained a significant predictor ($\beta = -0.330$, $p < 0.01$), whereas sex and HDRS-17 were not significant predictors.

This complementary analysis supports the interpretation that age-related changes in ventricular tracer dynamics are not solely attributable to ventricular enlargement

S4. Simulated Ventricular TACs Illustrating K_1 and k_2 Identifiability

To illustrate how K_1 and k_2 independently shape the ventricular time-activity curve (TAC), TACs were simulated using the S1TC model with a representative population arterial input function (median parameters across the cohort). In Figure S1(A), k_2 was held fixed at the cohort median value while K_1 was varied across the cohort's 10th to 90th percentile range; in Figure S1(B), K_1 was held fixed at the cohort median value while k_2 was varied across the same percentile range. Increasing K_1 primarily increased the magnitude of early-phase tracer uptake, with comparatively little effect on the shape of the late washout phase. Increasing k_2 primarily accelerated the rate of late-phase signal decline, with minimal effect on the early uptake magnitude. This simulation illustrates that K_1 and k_2 are governed by largely distinct portions of the dynamic curve, supporting their identifiability from the model fit.

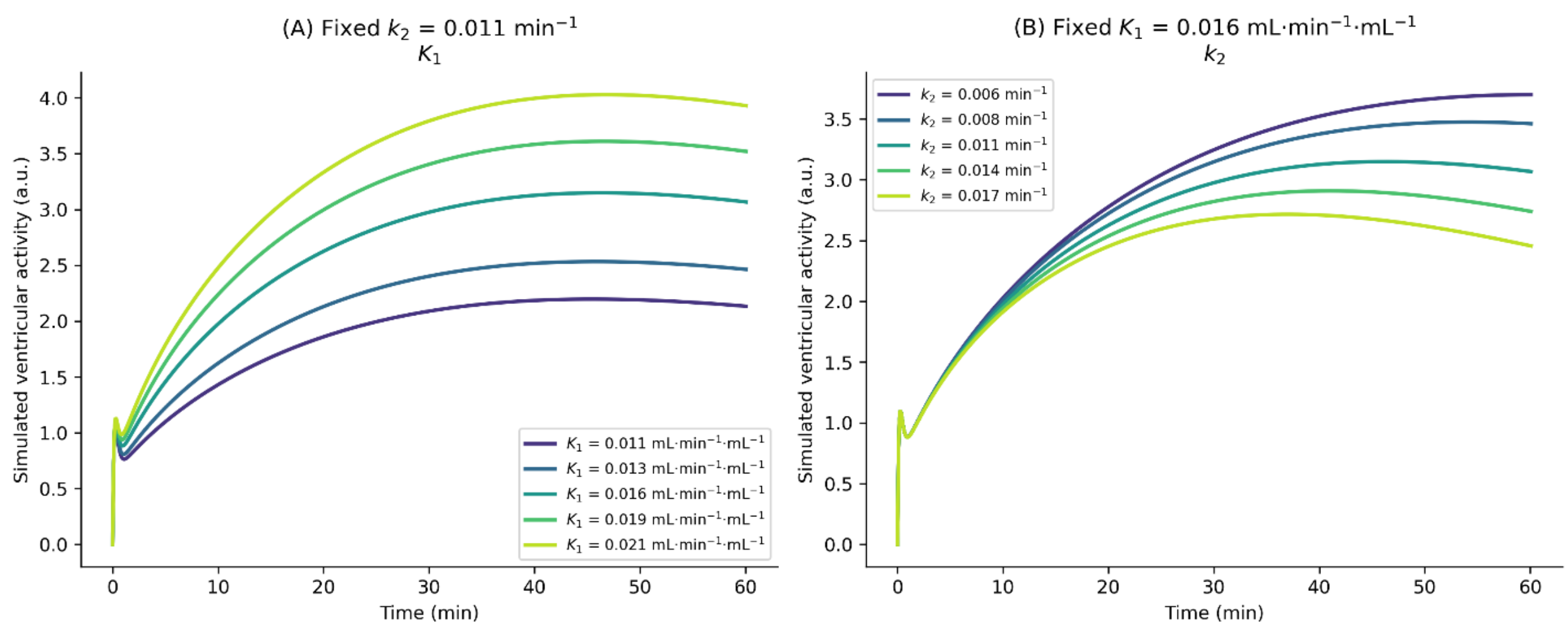

Figure S1. Simulated ventricular TACs from the S1TC model showing the effects of K_1 (A) and k_2 (B) on curve shape, using a representative population AIF. Each parameter varied across the cohort's 10th-90th percentile range while the other was held at the cohort median. Increasing K_1 raises overall curve magnitude, while increasing k_2 accelerates the late-phase decline.

S5. Erosion Sensitivity Analysis

The ventricular mask was eroded at 1, 2, 3, and 4 mm in MRI space. S1TC fitting, outlier handling, correlation, multivariable regression, and independence analyses were repeated at each level. Pre- and post-erosion ventricular volumes are summarized in Table 3 of the main manuscript.

The Pearson correlation between K1 and age was r = −0.700, −0.715, −0.707, and −0.629 at 1, 2, 3, and 4 mm erosion, respectively (all $p < 0.001$). The Pearson correlation between k2 and age was r = −0.712, −0.678, −0.630, and −0.513 (all $p < 0.001$). The K_1-k_2 correlation strengthened with erosion depth (r = 0.805, 0.862, 0.862, 0.887 at 1, 2, 3, and 4 mm, respectively).

In multivariable regression, age remained a significant predictor of K_1 across all erosion levels (β = −0.519, −0.527, −0.514, −0.437; all $p < 0.001$) and of k_2 across all erosion levels (β = −0.511, −0.480, −0.433, −0.323; all $p < 0.001$). Ventricular volume was also a significant predictor of both parameters at all erosion levels. HDRS-17 was a significant predictor of k_2 at 1, 2, and 3 mm erosion (β = 0.208, 0.155, 0.160; p = 0.004, 0.046, 0.049 respectively) but not at 4 mm (β = 0.113, p = 0.218). Sex was not a significant predictor of K_1 at any erosion level. For k_2, sex was not significant at all erosion levels, including 4 mm erosion (β = −0.121, p = 0.199).

In independence analyses, K_1 retained a significant independent age association after accounting for k_2 at all erosion levels (β = −0.338, −0.276, −0.294, −0.268; all $p < 0.001$). k_2's independent age association was present at 1 mm (β = −0.296, p = 0.004) but attenuated at 2 mm (β = −0.095, p = 0.291), 3 mm (β = −0.040, p = 0.662), and 4 mm (β = 0.104, p = 0.190).

At 4 mm erosion, mean post-erosion ventricular volume was 0.75 ± 1.22 cm^3, with 45 of 70 subjects retaining less than 0.50 cm^3 of ventricular ROI and 5 additional k2 outliers identified compared to shallower erosion levels. k_2 normality was restored after outlier removal, and the overall model R^2 decreased substantially (K_1: 0.597; k_2: 0.517) compared to shallower erosion levels. Per-subject ventricular volume trajectories across all four erosion levels are shown in Figure S2.

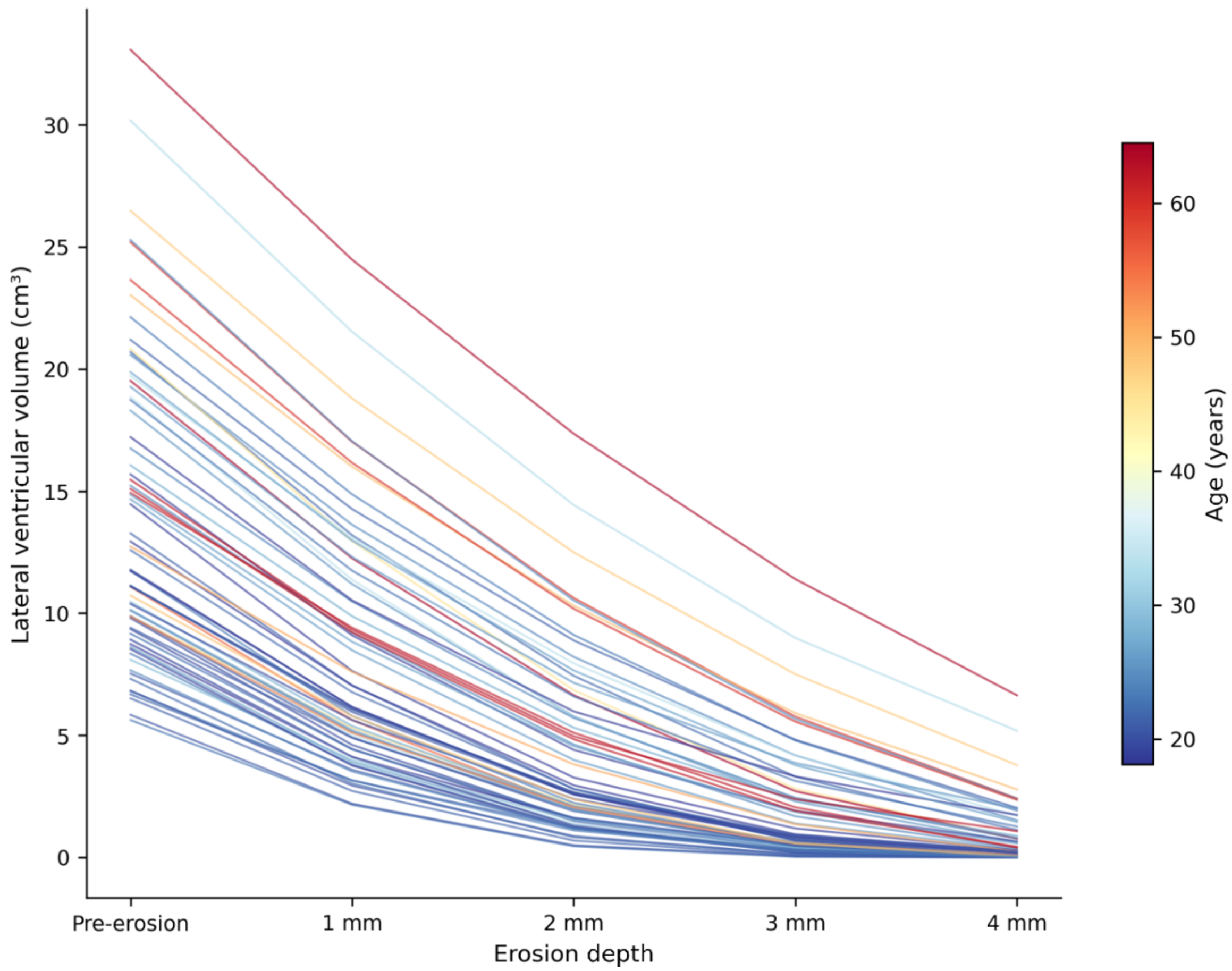

Figure S2. Per-subject lateral ventricular volume across erosion levels (1–4 mm), color-coded by age. Younger participants show the steepest volume reduction with increasing erosion depth.

S6. Choroid Plexus Sensitivity Analysis

Choroid plexus voxels were identified using an AI-based segmentation method (Li et al., 2025) and removed from the lateral ventricular ROI prior to TAC extraction and S1TC fitting. The analysis was repeated across all four erosion levels.

The mean number of choroid plexus voxels removed decreased substantially with erosion depth (477, 195, 46, and 10 voxels at 1, 2, 3, and 4 mm respectively), indicating that morphological erosion already removes the majority of choroid plexus voxels at shallower erosion depths.

Removal of choroid plexus voxels produced negligible changes in both K_1 and k_2 across all erosion levels. Mean K_1 differences were 4.8×10^{-5}, 6.7×10^{-5}, -3.6×10^{-5}, and -6.5×10^{-5} $mL \cdot min^{-1} \cdot mL^{-1}$ at 1, 2, 3, and 4 mm erosion respectively. Mean k_2 differences were −0.00051, −0.00062, −0.00075, and −0.00050 min^{-1}. These differences are negligible relative to the magnitude of the primary parameter estimates and are consistent across erosion levels.

These findings support the interpretation that residual choroid plexus signal does not materially influence the fitted kinetic parameters, and that the blood volume term in the S1TC model adequately accounts for residual vascular contamination within the ventricular ROI.

S7. Arterial Subgroup Analysis

To assess robustness to input function uncertainty, analyses were repeated in the 32 participants with arterial plasma samples. Both K_1 and k_2 were normally distributed (Shapiro-Wilk, K_1: p = 0.721; k_2: p = 0.910). One k_2 outlier was removed by IQR criterion (N = 31 for k_2 analyses). The Pearson correlation between K_1 and age was r = −0.753 (p < 0.001) and between k_2 and age was r = −0.708 (p < 0.001). In multivariable models adjusting for ventricular volume, sex, and HDRS-17, age remained a significant independent predictor of K_1 (β = −0.586, p < 0.001; R^2 = 0.735) and k_2 (β = −0.434, p < 0.001; R^2 = 0.788). The association between HDRS-17 and k_2 also remained significant (β = 0.364, p < 0.001). These findings are consistent with and numerically stronger than the full cohort results, confirming that the primary associations are not attributable to uncertainty introduced by venous-to-arterial input function scaling.
